# 

**Published:** 1875-04

**Authors:** 


### TABLE OF CONTENTS.

### ORIGINAL COMMUNICATIONS.

VALEDICTORY ADDRESS at Commencement Atlanta Medical College. *By J. H. Logan, M.D., Professor of Chemistry in the College*.......... 1

CLINICAL STUDIES with Large Non-Emetic Doses of Ipecacuanha: With a Contribution to the Therapeusis of Cholera. *By Alfred A. Woodhull, M.D., Asst. Surg. U. S. A. (Third paper.)*.......... 10

OBSTINATE VOMITING OF PREGNANCY Treated with Ipecacuanha. *By Meriweather Lewis, M.D., of Lenoir's, Tenn*.......... 28

### ATLANTA ACADEMY OF MEDICINE.

Connection between Varicella and Variola. Autopsy of case of Ovariotomy.—Muriated Tincture of Iron to Arrest Hæmorrhage from large Arteries.—Amputation of Thigh for Obstinate Disease of Bone ... 29

### CLINIC ATLANTA MEDICAL COLLEGE.

Diseases of Eye and Ear. *Prof. A. W. Calhoun, M.D*.......... 33

### OUR EXCHANGES.

The Anatomist's Ode to his Mistress.......... 39
Diphtheria.......... 40
Cancer—Practical Conclusions.......... 42
Digitalis in Heart Diseases.......... 42
Multiple Wedge in Dilating Urethral Stricture.......... 43
Warm Injections in Uterine Hæmorrhages.......... 45
Cincho-Quinine; Cough of Phthisis.......... 46
Utility of Medical Legislation.......... 47
Extraction of Placenta in Abortion.......... 48
Limit of Physician's Responsibility.......... 48
Premature Burial of Medical Papers.......... 49
Mistaken Pregnancy.......... 49
Cauliflower Excrescence—Portion of Peritoneum Removed.......... 50
Baltimore Quartet.......... 51
Etiology and Treatment of Pneumonia.......... 52
"Massage" in Sprains.......... 54
Enlarged Spleen.......... 55
Dangers of Intra-Rectal Examination.......... 56
Digitalis in Cardiac Dropsy.......... 56
Typhoid Fever.......... 57
Bromide Potassium to Supp rating Tumors.......... 58
Syrup of Brown Sugar an Antiseptic.......... 58
Delivery after Death—Life of a Six Months' Fœtus.......... 59
Habits of Cotton Worm.......... 60
Unauthorized use of M.D; Quinine in Epistaxis.......... 61
Recurrent Chronic Bronchitis; Railroad Surgeons.......... 61

### BIBLIOGRAPHICAL.

Pamphlets.......... 62

### EDITORIAL.

American Medical Association.......... 62

Meeting of State Societies.......... 63

Dr. J. G. Westmoreland on "Actions of Mercury".......... 63

Ladies and the JOURNAL.......... 63

Army Medical Staff.......... 64

Cincho-Quinine.......... 64

Married.......... 64

